# Lipase inhibitory activity assay for fermented milk

**DOI:** 10.1016/j.mex.2020.100999

**Published:** 2020-07-20

**Authors:** Ana María Gil-Rodríguez, Thomas P. Beresford

**Affiliations:** Teagasc Food Research Centre, Moorepark and Food for Health Ireland, Fermoy, Co. Cork, Ireland

**Keywords:** Lipase inhibitory activity, 4-nitrophenyl octanoate, Fermented milk, Two buffer system, P-nitrophenol

## Abstract

The lipase inhibitory activity method described here was developed to identify potential anti-obesity properties in milk fermented with different strains of lactic acid bacteria via inhibition of pancreatic lipase and a subsequent decrease in fat digestion and absorption in the gut. The method is based on the hydrolysis of 4-nitrophenyl octanoate by pancreatic lipase and the subsequent release of p-nitrophenol, a coloured product whose absorbance can be measured at 412 nm. Inhibition of lipase leads to a decrease in the amount of p-nitrophenol released and a subsequent reduction in the absorbance with respect to a 100% activity control. The assay was developed by adapting various methods previously described in published literature and includes modifications that are key to adapt the existing protocols to fermented milk samples, in particular the pH issues encountered when analysing acidic samples:

• A two buffer system is introduced to allow optimal pH control after addition of fermented milk samples with pH values between 3.5 and 6.5.
• A post-clarification filtration step is added for samples where turbidity remains after addition of the clarifying reagent for dairy products.
• An absorbance correction factor is calculated and applied to the samples to account for the reduction in the absorbance of p-nitrophenol caused by milk.

## Graphical abstract

**Figure:**
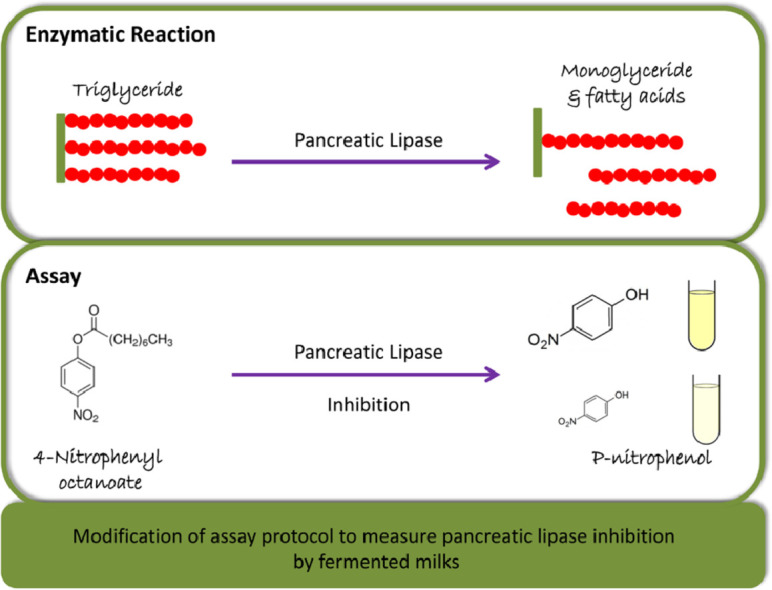

Specifications Table

**Table:** 

Subject Area:	*Agricultural and Biological Sciences*
More specific subject area:	*Food Science*
Method name:	*Lipase inhibitory activity assay for fermented milk*
Name and reference of original method:	*Methods for estimation of lipase Activity in milk* *Bendicho, S., Trigueros, M.C., Hernández, T., & Martin, O. (2001). Validation and comparison of analytical methods based on the release of p-nitrophenol to determine lipase activity in milk. Journal of Dairy Science, 84(7), 1590–1596.* *Estimation of lipase inhibitory activity in plat extracts* *Conforti, F., Perri, V., Menichini, F., Marrelli, M., Uzunov, D., Statti, G.A., & Menichini, F. (2012). Wild Mediterranean dietary plants as inhibitors of pancreatic lipase. Phytotherapy Research, 26(4), 600–604.*
Resource availability:	*N/A*

## Introduction

An excessive intake of calories combined with a lack of physical activity can lead to overweight and obesity, which are linked to multiple health complications such as metabolic syndrome, hypertension, type 2 diabetes mellitus and cardiovascular disease, among others [1,2].

In cases of low severity, digestive enzyme inhibitors are administered to obese subjects to reduce their calorie absorption from the diet. As obesity is usually associated with diets high in carbohydrates and/or fat, amylases and lipases are the main targets for this therapy [3,4]. In this context a “health through food” approach is an interesting option, as a food product might be better tolerated and accepted by the consumer than a pharmacological treatment [1,^4^,5].

Positive effects on obesity and lipid metabolism have already been reported with food products, edible plant extracts and strains of lactic acid bacteria [3,^4^,6]. The ability of the latter to regulate blood cholesterol levels via bile salt hydrolysis has been widely described [7], and recent studies report their capacity to specifically inhibit pancreatic lipase [8,^9^,10]. Milk fermented or supplemented with different LAB strains has been demonstrated to reduce body weight in obese mice [11], rats [12,13] and humans [8]. However, (and although lipase inhibitory activity has been studied in LAB strains) the inhibition of pancreatic lipase has not been considered as a potential mechanism of action of fermented milks with anti-obesity properties. As a consequence, a specific method to assay lipase inhibitory activity of fermented milk was not available.

The method described here was developed in the context of a study recently published by our group to screen fermented milk samples for lipase inhibitory activity with the objective of identifying fermentates with potential to be used to reduce body mass in overweight and obese subjects [14]. This method addresses the difficulties encountered when screening samples that vary significantly in their pH values, such as milk fermented by different strains of lactic acid bacteria. It also represents a useful tool to decipher the mechanisms of action of fermented milks with proven in vivo anti-obesity effect. To the best of our knowledge, the recent work by our group using the method described here is the first study where lipase inhibitory activity is reported in fermented milks.

## Method details

### Reagents and materials

• Reagents
  ○ Trizma base
  ○ Trizma-HCl
  ○ Dimethyl sulfoxide (DMSO)
  ○ Lipase from porcine pancreas, Type II
  ○ 4-nitrophenyl octanoate (NPC)
  ○ Clarifying reagent for dairy products (Sigma Aldrich, cat no. 00,467–500ML)
  ○ Orlistat (to use as positive control)
• Consumables
  ○ 5 mL tubes
  ○ 2.5 – 3 mL syringes
  ○ 0.2 µm PES filters
  ○ Plastic cuvettes for spectrophotometry
  ○ Capillary pistons for positive displacement pipette
• Equipment
  ○ Spectrophotometer
  ○ Vortex with adapter for at least 20 tubes
  ○ Water bath
  ○ Positive displacement pipette
  ○ Timer

## Preparation of buffers and solutions

### Tris–HCl buffer

The method described here is used to evaluate lipase inhibitory activity in fermented milk samples based on inhibition of pancreatic lipase. The pH of fermented milk can be as low as 3.5, and the buffers described in the cited literature (Tris–HCl between 0.05 and 0.06 M) [1,^15^,16] do not have the capacity to maintain the pH of the reaction mixture at the optimal for lipase activity (i.e., pH 8.5). In consequence, it was necessary to increase the concentration of the buffers to counteract the acidic pH of the samples. However, when the concentration of the buffer was increased to 0.15 M, the clarifying reagent for dairy products was unable to induce clarification of the reaction mixture (in agreement with [17]), but a single buffer at lower concentration was not capable of maintaining the pH of the reaction mixture at 8.5 when the most acidic samples were added. This led to the development of the two buffer system, in order to guarantee that the enzymatic reaction would take place at pH between 8.25 and 8.75 (described in detail in the below section “Method validation”).

The concentration of Tris–HCl used in this assay is 0.10 M, and two buffers at different pH values are required. In order to prepare the two buffers, it is necessary initially to prepare two solutions, one using Trizma-base and one using Trizma-HCl, both at 0.10 M. These two solutions are subsequently mixed to prepare the two buffers, until the desired pH values are reached (i.e., pH 8.5 and pH 9.5). It is important to take into consideration that the enzymatic reaction will take place at 37 °C and that the pH of Tris–HCl buffer decreases with increasing temperature. To compensate for this the pH of the solutions prepared at room temperature (between 19 and 21 °C) should be adjusted to 8.8 (for the pH 8.5 buffer) and 9.8 (for the pH 9.5 buffer). Once adjusted and stabilised, it is recommended to measure the pH of an aliquot heated to 37 °C.

It was observed that the pH of the buffers could change after being adjusted to the desired value. Thus, it is recommended to prepare the buffers at least one day before performing the lipase inhibition assay and leave them at room temperature overnight. The pH should be checked the following morning and re-adjusted, if necessary, by adding Trizma-base or Trizma-HCl solution. Once the desired pH is reached and the buffers are stable, it is recommended to filter-sterilize the buffer solutions into sterile bottles to increase their shelf-life.

### Pancreatic lipase

The solution containing the enzyme can be stored for long periods at −20 °C. Therefore, it is recommended to prepare a concentrated stock solution at 50 mg/mL, transfer 100 µL aliquots into 1.5 mL centrifuge tubes and freeze immediately. In order to maximize lipase activity, it is recommended to prepare the solution as follows:

• Label 1.5 mL centrifuge tubes (20 tubes).
• In a small glass beaker add 2 mL Tris–HCl buffer pH 8.5.
• Place a stirring magnet inside the beaker.
• Fill an empty pipette-tip box (or similar) with ice and place the beaker in its center.
• Using a small vessel (e.g., 2 mL screw cap tube) weigh 100 mg lipase powder.
• Place the box containing the ice and the beaker on a stirring plate and activate the stirrer at low speed to avoid the formation of bubbles, as this can provoke denaturation of the enzyme.
• Add the 100 mg lipase powder slowly to the buffer. The powder should be added in small amounts to facilitate the complete dissolution of the enzyme and avoid the formation of aggregates.
• Once all the lipase powder is added leave stirring for 5 min.
• While continuing to stir, distribute the lipase solution in 100 µL aliquots into the 1.5 mL centrifuge tubes (maintain in ice).
• Freeze at −20 °C.

Before each assay, add 900 µL Tris–HCl buffer at pH 8.5 to one 100 µL pancreatic lipase stock solution aliquot (still frozen) and mixed by gently pipetting. Once the ice is thawed, invert the tube gently several times, do not vortex. The work solution (5 mg/mL) is ready to use and any unused material should be discarded once the assay is completed.

### 4-nitrophenyl octanoate (NPC)

The concentration of 4-nitrophenyl octanoate used is 5 mM in DMSO. As for pancreatic lipase, it is recommended to prepare a stock solution 10 times more concentrated (i.e., 50 mM) from which the working solution can be easily prepared. As NPC is a liquid, the volume needed was calculated using its density (1.095 g/mL) to facilitate the preparation of the stock solution: e.g., to prepare 5 mL of 50 mM stock solution, 66.325 mg are needed, which divided by 1.095 gives 60.57 µL. The concentrated stock solution was aliquoted as described for the pancreatic lipase stock solution. To protect the reagent from the light, brown 1.5 mL centrifuge tubes were used to store the solution.

This reagent should not be stored at temperatures below 0 °C, and therefore, this solution was kept at 4 °C. At this temperature, DMSO is frozen and the solution should be left to thaw at room temperature for at least 20 min before starting the assay. To obtain the work solution, add 900 µL DMSO into one 100 µL aliquot of NPC stock solution.

### Orlistat

Orlistat (tetrahydrolipstatin) is a potent lipase inhibitor used to treat overweight and obese subjects [3] and is commonly used in lipase inhibition assays as a positive control [1,^6^,^16^,18]. In this study, orlistat is used as a control for assay validation. The concentration used is 10 mg/mL final concentration, i.e., in the reaction mixture. The solution is prepared by dissolving 5.2 mg of orlistat into 1 mL of DMSO.

## Procedure

### Reaction phase

1. Label 5 mL tubes in duplicates. A maximum of 20 tubes are used at a time, which allows analysis of two controls and three samples.
  a. B: blank
  b. C: control (100% activity)
  c. O: orlistat control (when applicable)
  d. BM: blank for non-fermented milk control
  e. M: non-fermented milk control
  f. Tubes for 3 samples and their respective blanks in duplicates
2. Add Tris–HCl buffer to each one of them (see Table 1 for volumes).

**Table 1:** Reagent volumes added to each reaction tube.

Reagent	B	C	O	BM	M	Samples
Tris–HCl	2.55 mL	2.50 mL	2.45 mL	2 mL	2 mL	2 mL
Milk	—	—	—	500 µL	500 µL	
Fermented milk	—	—	—	—	—	500 µL
DMSO	—	—	—	50 µL	—	—
NPC	50 µL	50 µL	50 µL	—	50 µL	50 µL
Orlistat	—	—	50 µL	—	—	—
Lipase	—	50 µL	50 µL	50 µL	50 µL	50 µL
Final volume	2.60 mL	2.60 mL	2.60 mL	2.60 mL	2.60 mL	2.60 mL
3. Add 500 µL non-fermented milk to tube M or fermented milk to sample tubes (see Table 1).
4. Add 50 µL NPC to reaction tubes. Replace with DMSO in blanks.
5. Add 50 µL lipase solution.
6. Vortex 2 min (using an adapter, all tubes can be vortexed simultaneously).
7. Incubate in water bath at 37 °C
8. Start timer (30 min).

### Clarification and reading phase

1. Add 1 mL clarifying reagent for dairy products to each tube.
2. Mix gently to avoid foaming. Do not vortex.
3. Incubate 3 min in water bath at 37 °C.
4. Transfer 2.7 mL into a cuvette. If turbidity remains after incubation with the clarifying reagent, filter through a 0.2 µm PES filter.
5. Measure absorbance at 412 nm.

*Note:* to guarantee accuracy we strongly recommend the use of a positive displacement pipette when manipulating coagulated milk samples and clarifying reagent for dairy products.

For every set of assays performed, a 100% activity control and a non-fermented milk control (C and M respectively in table 1) must be included (the non-fermented milk control could potentially be replaced with an orlistat control). To keep processing times to a minimum, a small number of samples (three in this case) should be processed with each assay, and this number should be kept constant to reduce inter-assay variability.

After obtaining the absorbance measurements, lipase inhibitory activity is calculated as percentage of activity with respect to the 100% activity control using formula 1:

$$Lipase\;inhibitory\;activity\;\left(\%\right)=100-\;\left(\frac{Abs_{S}-Abs_{SB}}{Abs_{RC}-Abs_{B}}\;\times \;100\right)$$

*Formula 1:* Equation for the calculation of lipase inhibitory activity based on absorbance values. *Abs_S_*: absorbance of the sample; *Abs_SB_*: absorbance of the blank of that sample; *Abs_RC_*: absorbance of the 100% activity control; and *Abs_B_*: absorbance of the blank of this control.

Before calculating lipase inhibitory activity, a correction factor of 5.71% should be applied to the absorbance values obtained for the samples, as the addition of milk decreases the absorbance of the released p-nitrophenol in that proportion (described in detail in the below section “Method validation”).

*Note:* a control with a known level of lipase inhibition should always be included to monitor the results obtained with each experiment (e.g., control milk or orlistat at a constant concentration). In addition, the absorbance of the blank tubes can be used to monitor assay conditions, as alterations in the buffers will cause the blank values to vary.

## Method validation

### Definition of optimal pH for lipase activity

**Fig. 1:**
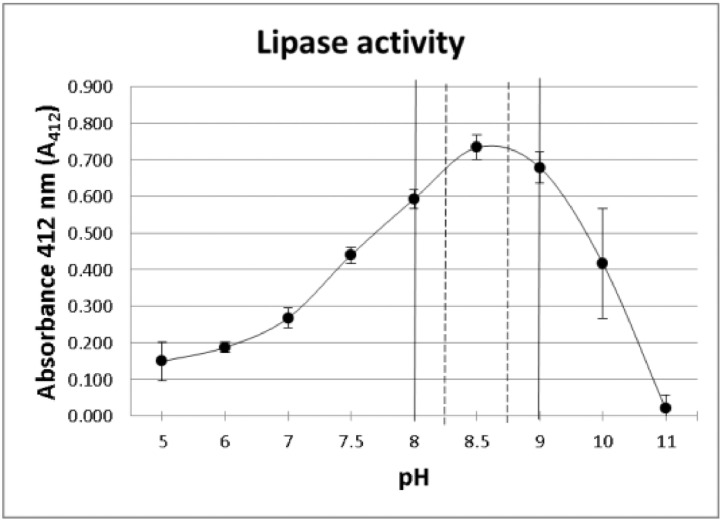
Lipase activity curve at various pH values (5 to 11). At pH 10 and 11 NPC is spontaneously hydrolysed and the yellow color appears even when the enzyme is absent, *i.e.*, the absorbance of both blank and assay tube were very high. The optimal pH interval obtained from literature is marked by vertical continuous lines. The proposed narrower optimal pH interval for the current assay is highlighted with dashed lines.

In previous methods, the pH of the buffer used to determine lipase activity or inhibition was 8.5 [1,^15^,^16^,18]. However, a wider range of pH (between 8.00 and 9.00) is considered by other authors as optimal for lipase activity [19,20]. In order to establish the optimum pH range for the enzyme and assay conditions used in the current study a lipase activity curve using buffers at different pH values was prepared (Fig. 1). Briefly, a set of Tris–HCl buffers at nine different pH values (between 5 and 11) was prepared by mixing Trizma-base and Trizma-HCl solutions as previously described. Lipase inhibitory activity was conducted as described in the previous “Method details” section using the buffers at different pH values (no milk was added here). Experiments were performed in duplicates and repeated three times.

It can be observed that the optimum pH under the experimental conditions tested is 8.5, the value taken from literature for the assay [1,^15^,^16^,18]. Additionally, the activity seems to be higher and more stable between pH 8.5 and 9.0 than between pH 8.0 and 8.5, where a higher variation is observed. According to these results, if the pH of the reaction mixture on a particular sample decreases from 8.5 to 8.0, a reduction in lipase activity will be observed, and this decrease in activity could be wrongly interpreted as lipase inhibition. Thus, in order to guarantee the accuracy of the method, a new pH interval for optimal lipase activity was defined at 8.25–8.75.

### Development of the two buffer system

As described in previous sections, the addition of an acidic sample to the reaction vessel (500 µL to a final volume of 2.60 mL) can cause a decrease in the pH of the reaction mixture from 8.5 to 5.97 when the pH of the sample is 3.63 and the buffer used is Tris–HCl at 0.05 M (Table 2), causing a drastic drop in lipase activity. In order to decrease the pH change of the reaction mixture following addition of the sample, the used buffer (Tris–HCl) was prepared at higher concentrations (0.10 and 0.15 M), so as to increase its buffering capacity. Additionally, the pH reduction as a consequence of temperature increase (from room temperature to 37 °C) was also considered and, therefore, the buffers were prepared at pH 8.8 at room temperature, which descends to a pH value of 8.5 after warming it to 37 °C. The pH values of the different buffers after addition of non-fermented skim milk (NFSM) and a sample at pH 3.63, respectively, were measured (Table 2).

**Table 2:** pH values of Tris–HCl at different concentrations and pH values after the addition of NFSM and a sample of fermented milk at pH 3.63. The values obtained at both room temperature (21 °C) and at 37 °C, the temperature at which the lipase inhibitory activity assay is performed, are presented. Note that at 37 °C only the buffer at concentration 0.15 M can maintain the pH above 8 when the sample at pH 3.63 is added. Tris–HCl 0.05 M pH 8.5 is the buffer used in other published methods.

Tris–HCl concentration	21 °C			37 °C		
	pH buffer	pH NFSM	pH 3.63	pH buffer	pH NFSM	pH 3.63
0.05 M pH 8.5	8.60	8.42	6.35	8.20	8.27	5.97
0.10 M pH 8.5	8.62	8.58	7.96	8.32	8.38	7.72
0.10 M pH 8.8	8.89	8.84	8.18	8.65	8.64	7.93
0.15 M pH 8.8	8.87	8.93	8.44	8.72	8.69	8.17

When buffer concentration was > 0.10 M, addition of clarifying reagent for dairy products did not clarify the sample. Therefore, a set of Tris–HCl buffers was prepared at 0.10 M with higher pH values. The pH variations in these buffers were assessed after addition of milk acidified with lactic acid to pH 3.5, 4.0, 4.5 and 5.0, as well as milk without acidification (pH 6.5). The results are displayed in Table 3.

**Table 3:** Changes in the pH of Tris–HCl 0.10 M at different initial pH values after addition of control milk acidified with lactic acid to pH between 3.5 and 5.0, and without acidification (NFSM). Highlighted in bold are the pH values of the two selected buffers after addition of the control milks: Tris–HCl 0.10 M 8.5 selected for samples with pH>4.5 and Tris–HCl 0.10 M 9.5 selected for samples with pH≤4.5. All measurements were taken at 37 °C.

Buffer	pH	pH after addition of acidified milk				
		3.5	4.0	4.5	5.0	NFSM
Tris–HCl 0.10 M 8.5	8.55	7.90	8.13	8.30	**8.46**	**8.59**
Tris–HCl 0.10 M 9.0	8.99	8.11	8.28	8.43	8.60	8.82
Tris–HCl 0.10 M 9.5	9.49	**8.27**	**8.44**	**8.62**	8.84	9.10
Tris–HCl 0.10 M 10.0	10.03	8.36	8.57	8.78	8.99	9.35

### Validation of the two buffer system

**Fig. 2:**
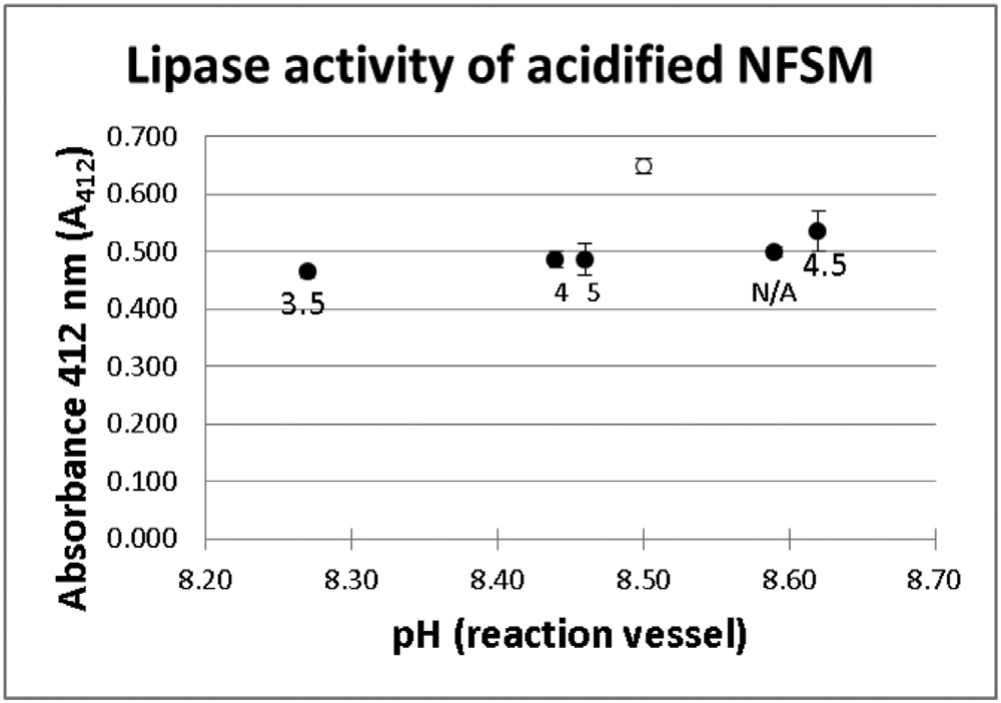
Absorbance of NFSM acidified with lactic acid to different pH values (3.5, 4.0, 4.5 and 5.0). The white circle indicates the absorbance of the 100% activity control, *i.e.*, absorbance of the reaction without addition of milk. The number below each point indicates the pH value of the milk added. N/A: non acidified milk.

In order to validate the pH control achieved with the newly developed two-buffer system and that the activity of the more acidic samples was not due to a decrease in the pH of the reaction mixture below 8.25, the control milks acidified to different pH values with lactic acid used in the previous section were subject to the lipase inhibitory activity test using the two-buffer system (Tris–HCl 0.10 M pH 9.5 for the controls at pH 3.5, 4.0 and 4.5 and Tris–HCl 0.10 M pH 8.5 for the control at pH 5 and the control without pH adjustment). The absorbance values obtained for each of the acidified milks after blank subtraction are presented in Fig. 2. Absorbance values for all tested milks were very close to 0.500, regardless of the pH of the reaction vessel (average 0.494 ± 0.023).

**Fig. 3:**
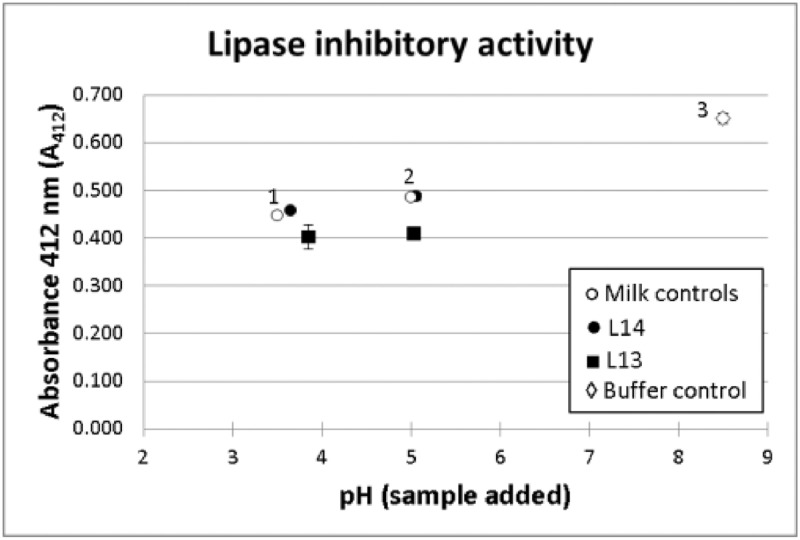
Lipase inhibitory activity of L13 and L14 at their original pH and at pH 5.0 (absorbance values presented). The white circles indicate the different milk controls: 1) non-fermented milk acidified to pH 3.5; 2) non-fermented milk acidified to pH 5.0; 3) Control 100% activity, *i.e.*, no milk is added.

As further validation of the protocol; lipase activity was measured in two fermented milk samples (L13 and L14) randomly selected, with pH values of 3.85 and 3.65, respectively. An aliquot of each was taken and its pH increased to 5 using 2.5 M NaOH. These samples were then processed, both at the original pH value and at pH 5 using Tris-HCl 0.10 M pH 9.5 and Tris-HCl 0.10 M pH 8.5, respectively. For the two samples, the absorbance obtained was at the same level both at the original pH value and when the pH was increased to 5 (Fig. 3). For L14 the activity is, in both cases, at the same level as the non-fermented control milk, thus indicating a lack of inhibitory activity beyond the interference due to the addition of milk solids to the reaction vessel. For L13, however, a lower activity is reported, with a lipase inhibition of 33.17 ± 4.06% in the sample at the original pH and 33.75 ± 1.05% when the pH was adjusted to 5. The NFSM control gave a basal inhibition level of 23.60 ± 2.03%.

Overall, these results suggest that the activity of the same sample, including non-fermented control milk, remains constant when the pH is changed. This indicates that i) the interference or inhibition seen is pH-independent and that ii) the possible effect of sample pH on lipase activity is removed with the two buffer system, thus allowing an optimal pH control in the reaction vessel.

### NFSM basal inhibition level and absorbance correction factor

In the previous validation steps (see Fig. 2); a reduction in the absorbance was observed when NFSM was added to the reaction. To investigate whether this was due to interference with the reaction itself or with the absorbance of p-nitrophenol, the assay was performed as previously described except that NPC was replaced with a 5 mM solution of p-nitrophenol to simulate 100% hydrolysis of 4-nitrophenyl octanoate by lipase (Table 4).

**Table 4:** Reagents added to each reaction tube. **B:** Blank without milk; **p-NP:** p-nitrophenol 100% absorbance; **BM:** blank with milk; ***M*** **+** ***p*-NP:** 100% absorbance of p-nitrophenol in presence of milk.

Reagent	B	p-NP	BM	*M* + *p*-NP
Tris–HCl	2.50 mL	2.50 mL	2 mL	2 mL
Milk	—	—	500 µL	500 µL
DMSO	50 µL	—	50 µL	—
p-nitrophenol	—	50 µL	—	50 µL
Lipase	50 µL	50 µL	50 µL	50 µL

Final volume	2.60 mL	2.60 mL	2.60 mL	2.60 mL

A decrease of 5.96% in the absorbance of p-nitrophenol was observed when milk was added to the reaction mixture with respect to the reaction where no milk was added. This experiment was repeated a further seven times and the values obtained were used to calculate an average value of 5.74% (Table 5), which was subsequently used as a correction factor to the absorbance obtained for the samples and the milk controls.

**Table 5:** Absorbance values from the experiments performed to calculate the reduction in the absorbance of p-nitrophenol caused by the addition of milk to the reaction mixture.

p-NP	p-NP + milk	Absorbance reduction (%)
0.929	0.874	5.96
0.930	0.875	5.91
0.927	0.870	6.15
0.930	0.876	5.81
0.861	0.813	5.57
0.937	0.876	6.51
0.920	0.878	4.57
0.933	0.882	5.47

Average		5.74

This correction factor, however, did not fully account for the reduction in absorbance observed after addition of milk, which was of around 24% after absorbance correction. It was considered that the addition of milk solids to the reaction was responsible for this basal level of activity.

## Final method flowchart



## Declaration of Competing Interests

The authors declare that they have no known competing financial interests or personal relationships that could have appeared to influence the work reported in this paper.
